# A Case of Obstinate Costiveness

**Published:** 1781-05

**Authors:** 


II. *A Cafe of obftinate Coftivenefs. Communicated by* John Elliott, M. D. *Read May* 14, 1781.

ON the 21ft of November laft I was confulted by a young woman, twenty years of age, who, till then, had enjoyed an uninterrupted ftate of good health. She was well-made, and naturally lively, and feemingly rather of a robuft conftitution.

She

She complained of pains in her legs, thighs, back, and head; lownefs of fpirits, ficknefs at the ftomach, and lofs of appetite. Her urine was in a natural quantity, but high-coloured; her pulfe quicker than ufual, beating about ninety ftrokes in a minute; her tongue flightly incrufted, and fhe complained of thirft. Tho' never before fubject to coftivenefs, fhe had now, I found, been eleven days without going to ftool; but as fhe had ate but little during that time, fhe had afcribed her want of ftools to that circumftance. She had no yellownefs of the fkin, or tunica conjunctiva; no fymptom that indicated an obftruction of the biliary ducts.

I thought it neceffary to begin by procuring ftools; and as in general fhe had experienced that the mildeft purgative was fufficient to produce this effect, I contented myfelf with prefcribing fix drachms of Glauber's falt, and an equal quantity of manna, to be diffolved in two ounces of water, with the addition of fix grains of the powder of jalap. The patient fwallowed this draught, and drank liberally of weak broth and other diluting liquors to promote its operation, but without effect. After a few hours the fame draught was repeated, with the addition of fifteen grains of the powder, and and two drachms of the tincture of jalap. This was not more fuccefsful than the laft, nor did it occafion any vomiting, or much inclination to vomit, or any confiderable pain in the ftomach. In a few hours after fhe had taken it her thirft increafed, her head-ache grew worfe, and I advifed the ufe of a purgative and emollient glyfter, at the fame time directing two table fpoonfuls of a purging mixture to be taken every two hours. The mixture confifted of two ounces of Glauber's falt, diffolved in eight ounces of infufion of fena.—The glyfter brought away with it a confiderable portion of hardened fœces, but the mixture produced no evacuation. During all this time fhe took nourifhing things occafionally, but never brought any thing from off her ftomach, and feldom complained of being fick.

*Nov.* 22.—Her abdomen felt confiderably diftended, and was fomewhat painful when touched. Her pulfe was quicker and fuller than yefterday.—I advifed the taking away eight ounces of blood, and directed the glyfter to be twice repeated in the courfe of the day. They each remained with her about an hour, and produced fimilar effects to the firft; but with this difference, that they had occafioned confiderable pain pain during their ſtay. She continued the uſe of the mixture, with the addition of *Ol. Ricini*, but without any purgative effect.

*Nov.* 23.—The patient's belly was leſs painful, and not more tumid than yeſterday. — A repetition of the glyſter occaſioned more pain than yeſterday, and brought away ſome fœces with it.—It was repeated again in the evening, and an hour previous to its being injected, ſhe took fifteen grains of the *Extract. Cathart.* and two grains of the *Extract. Thebaic.* made into pills. Theſe prevented the painful effect of the glyſter, but procured no evacuation. She continued the uſe of the mixture.

*Nov.* 24.—The patient had ſlept three hours, and was much refreſhed.—She had voided a good deal of high-coloured urine.—She continued the uſe of the mixture, and repeated the pills and glyſters twice in the courſe of the day.—At night ſhe tried the warm bath.

*Nov.* 25.—The patient was nearly in the ſame ſtate as the day before, only that her pulſe was quickened to one hundred, and her thirſt was more increaſed. I therefore directed manna, inſtead of Glauber's ſalt, to be diſſolved in the mixture, and adviſed her to eat tamarinds, ſtewed pruants, &c.—Remembering to have read in the *Edinburgh Medical Eſſays* of the efficacy of cold cold bathing in obftinate conftipation, I directed cold water to be thrown on the patient's feet and legs, but this had no better effect than the warm bath tried the night before. The cafe feemed favourable for the trial of quickfilver, but I was fearful it might do irreparable mifchief by its weight and ftimulus in cafe it did not pafs.—As there were at prefent no fymptoms that indicated any inflammation of the bowels, I determined to perfevere in the frequent ufe of glyfters and mild evacuants, and therefore ventured to prefcribe the former without the previous ufe of the pills; but the pain was very confiderable and of long duration, fo that we were obliged to have recourfe to them again, and they had the fame good effect as before. In this ftate the patient continued till the 30th, when fhe had a confiderable evacuation by ftool, and the day following another, but not without a repetition of the glyfters and pills, to the ufe of which we found it neceffary to recur occafionally till the 14th of December, when fhe began to evacuate her fœces in a natural manner. She foon recovered, and is now in perfect health.

As I find it difficult to account fatisfactorily for all the phenomena of the above cafe, I fhall content myfelf with having related the facts as

 they they occurred to me, without attempting to reaſon on them. If they ſhould meet with the approbation of the learned Society to whom I have the honour to preſent them, my end will be fully anſwered.

*Carnaby Market, London,*
May 8th, 1781.

